# Modelling of chemotactic sprouting endothelial cells through an extracellular matrix

**DOI:** 10.3389/fbioe.2023.1145550

**Published:** 2023-06-08

**Authors:** Josep Ferre-Torres, Adria Noguera-Monteagudo, Adrian Lopez-Canosa, J. Roberto Romero-Arias, Rafael Barrio, Oscar Castaño, Aurora Hernandez-Machado

**Affiliations:** ^1^ Department of Condensed Matter Physics, University of Barcelona (UB), Barcelona, Spain; ^2^ Electronics and Biomedical Engineering, University of Barcelona (UB), Barcelona, Spain; ^3^ Biomaterials for Regenerative Therapies, Institute for Bioengineering of Catalonia (IBEC), The Barcelona Institute of Science and Technology (BIST), Spain; ^4^ Institute for Research in Applied Mathematics and Systems, National Autonomous University of Mexico , Mexico City, Mexico; ^5^ Institute of Physics, National Autonomous University of Mexico, Mexico City, Mexico; ^6^ Institute of Nanoscience and Nanotechnology (IN2UB), University of Barcelona (UB), Barcelona, Spain

**Keywords:** extracellular matrix, angiogenesis, chemotaxis, endothelial cells, biomimmetic, phase field, mathematical models, *in silico* model

## Abstract

Sprouting angiogenesis is a core biological process critical to vascular development. Its accurate simulation, relevant to multiple facets of human health, is of broad, interdisciplinary appeal. This study presents an *in-silico* model replicating a microfluidic assay where endothelial cells sprout into a biomimetic extracellular matrix, specifically, a large-pore, low-concentration fibrin-based porous hydrogel, influenced by chemotactic factors. We introduce a novel approach by incorporating the extracellular matrix and chemotactic factor effects into a unified term using a single parameter, primarily focusing on modelling sprouting dynamics and morphology. This continuous model naturally describes chemotactic-induced sprouting with no need for additional rules. In addition, we extended our base model to account for matrix sensing and degradation, crucial aspects of angiogenesis. We validate our model via a hybrid *in-silico* experimental method, comparing the model predictions with experimental results derived from the microfluidic setup. Our results underscore the intricate relationship between the extracellular matrix structure and angiogenic sprouting, proposing a promising method for predicting the influence of the extracellular matrix on angiogenesis.

## Introduction

The ability of cells to migrate is essential for fundamental biological processes involving vasculature development, angiogenesis and wound healing Qiu and Hirschi (2019); Friedl and Gilmour (2009). Specifically, migrating endothelial cells (ECs) are crucial in the formation of a functioning vascular network and angiogenesis. EC constitutes the inner cellular lining of capillaries, arteries and veins. ECs need to adapt, forming new vessels responding to tissue injury or lack of nutrient conditions. To produce a functional vasculature, growth and transcription factors interplay to balance angiogenesis and EC migration Jain (2003); Krüger-Genge et al. (2019); Carmeliet and Jain (2011).

Sprouting angiogenesis, or the penetration of vessels into avascular tissue, is critically dependent on the interactions between ECs, pericytes, and the extracellular matrix (ECM) Marchand et al. (2019); Blocki et al. (2018). The ECM plays an integral role in supporting and guiding the cellular sprouting process. Initially, endothelial cells extend filopodia to probe the surrounding ECM environment, guided by gradients of bioactive molecules De Smet et al. (2009). The ECM, in turn, provides a physical scaffold for these exploratory cells and presents a rich source of sequestered growth factors that can be liberated by enzymatic degradation Schultz and Wysocki (2009). Key among the molecular regulators of angiogenic sprouting is the vascular endothelial growth factor (VEGF), which promotes the proliferation and migration of endothelial cells. In addition to VEGF, the Delta-like ligand 4 (Dll4)-Notch signalling pathway plays a critical role in regulating the selection and behaviour of the sprouting cells Pitulescu et al. (2017). The dynamic interplay between cells and the ECM, conditioned by the liberation of sequestered proteins and bioactive molecules, lies at the heart of angiogenic sprouting. This complex relationship not only shapes cellular behaviours during angiogenesis but also contributes to the stabilisation and maturation of the emerging vascular network. Furthermore, integrins, a family of cell surface receptors, are central to cell-ECM interactions, mediating cell adhesion to the ECM and transducing biochemical and biomechanical signals from the ECM into the cell Kanchanawong and Calderwood (2023). Recognizing the pivotal role of integrins and bioactive molecules in mediating the dynamic interplay between cells and the ECM, the significance of the ECM extends beyond a mere structural scaffold. Indeed, it serves as an active participant in cellular processes, functioning as a complex and adaptable microenvironment. Through the ECM, cells can migrate, proliferate, sense, communicate with other cells, and even remodel this same ECM in a sophisticated and spatio-temporal synchronisation Lutolf and Hubbell (2005). The reciprocal cell-matrix interactions and the different species diffusing along the ECM determine many of the cell behaviour. Because of the strong interaction between cells and their surrounding, the fabrication of artificial ECM has become of tremendous interest and challenge.

Hydrogels, in many forms, are widely used as extracellular matrix materials for *in vitro* experiments Vila et al. (2020); Krishnamoorthy et al. (2019); Blache and Ehrbar (2018); the use of hydrogels is so far the only option, given their biocompatibility, softness and water content Caló and Khutoryanskiy (2015). Nowadays, tissue engineering can promote angiogenic growth factor release and vascularisation to irrigate tissues. However, still, there is a lack of control and synchronisation between fabricated ECMs, vasculature structures and cell fates. Therefore, theoretical studies and computational modelling add value to understanding and elucidating how the structure of hydrogels, or artificial extracellular matrices, can influence cell dynamics Vats and Benoit (2013); Ma et al. (2021); Zhang K. et al. (2021); Elosegui-Artola (2021). Fibrin-based hydrogels have been used as ECM analogues thanks to their feasibility of tuning their mechanical properties by simply changing concentrations or gelation conditions.

**FIGURE 1:**
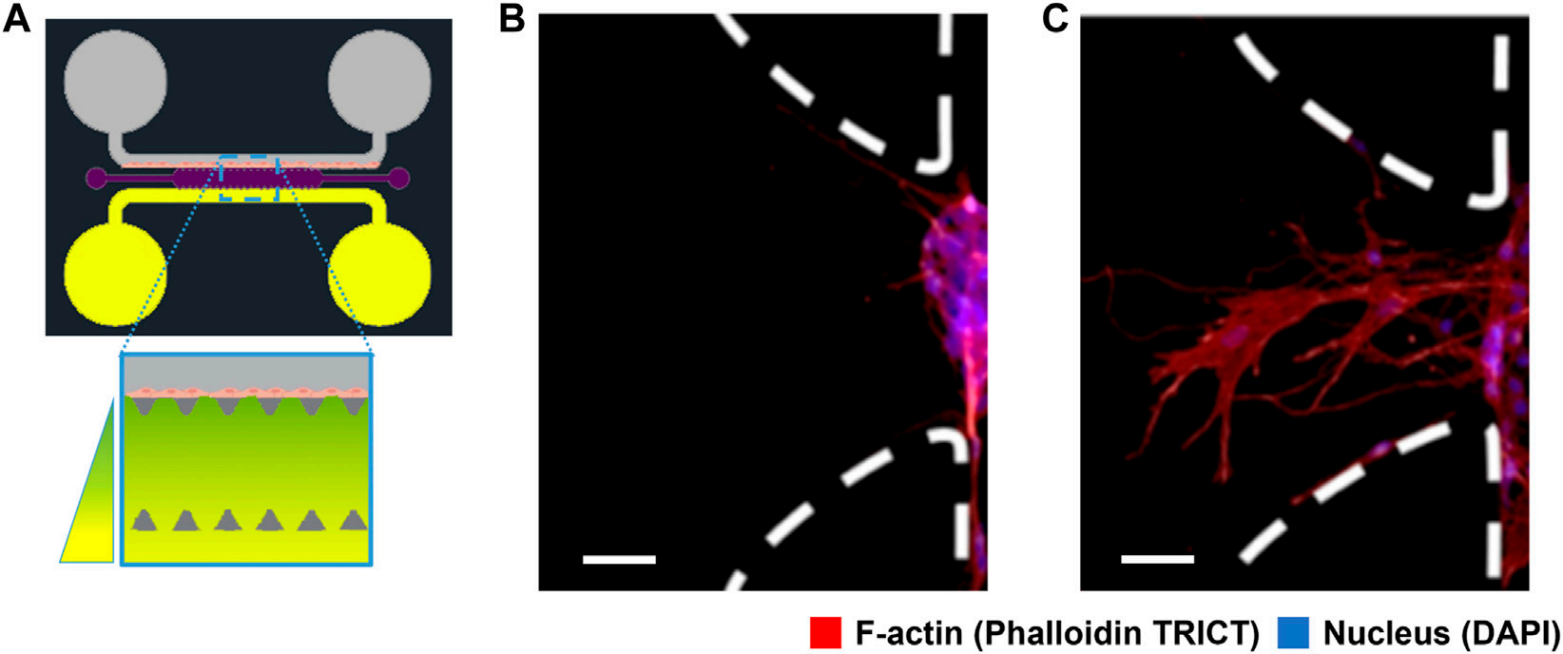
Schematic representation of the experimental setup and results of cell migration. **(A)** Scheme of the microfluidic platform design for angiogenic cell migration within the central chamber (1300 *μm* wide, 8800 *μm* long and 150 *μm* high) filled up with fibrin-based hydrogel (violet). Two lateral channels are connected to the central chamber through a trapezoidal structure (dark grey) with deposits at their ends. One side channel contains rat endothelial progenitor cells (rEPC) (grey), and the opposite side channel is connected to reservoirs with angiogenic factor concentration (vascular endothelial growth factor, VEGF) (yellow). **(B)** Fluorescence microscopy image of the cells inside the microfluidic system without angiogenic factor after 48 h. **(C)** Fluorescence microscopy image of the cells after 48 h under the influence of the angiogenic factor gradient. Endothelial cells migrate from an initial endothelium or isle, at one side of the channels, towards the higher concentration of VEGF, forming a finger-like structure. The scale bar in **(B,C)** represent 50 *μm*. Cells were stained for F-actin (red), and cell nuclei (blue). Images **(B,C)** depict the trapezoidal structure marked with white dashed lines, rotated 90° from image **(A)**.

In this study, we have performed experiments to study the advancement of ECs through a large-pore, low-concentration fibrin-based hydrogel matrix due to a chemotactic factor gradient concentration. This hydrogel matrix generates a porous landscape for cells to migrate, replicating their natural environment, i.e., acting as an ECM. The mechanical properties of the hydrogel are characterised at the macro-scale in the Supplementary Material, *“Characterisation of the hydrogel structure”*. In (Figure 1A) we show the experimental design. The microfluidic platform consists of a central chamber for the hydrogel and two side channels, with two reservoirs each. Reservoirs are filled up with different cell media volumes to generate flow inside the side channels due to the difference in hydrostatic pressure. At one side of the central chamber, we have grown an endothelium using rat endothelial progenitor cells (rEPCs). On the other side-channel, we placed a chemotactic factor, VEGF, that diffuses through the ECM. We see in (Figures 1B, C) the initial distribution of cells and their response to the chemotactic factor gradient after 48 h.

**FIGURE 2:**
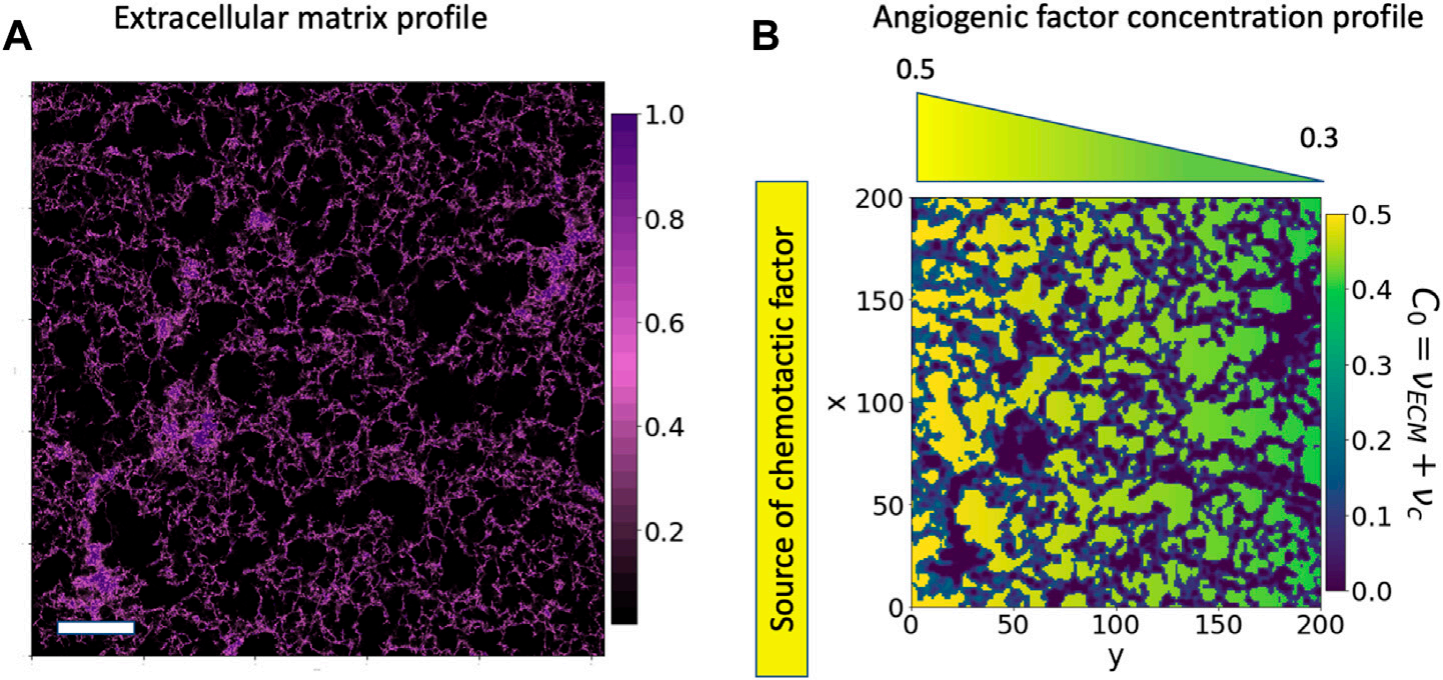
Cell environment and modelled cell environment: confocal microscopy image of a section of the 3-D hydrogel using a Z-stack. **(A)** Confocal microscopy image of a region of 775* μm* ^*2*^ of fibrin-based hydrogel used for cell migration; the image has been generated by NHS-rhodamine covalently linked to amine groups included in fibrin. *In-vitro* experiments use this hydrogel to study endothelial cell migration and angiogenesis. The colour scale represents the hydrogel concentration present at each pair of coordinates. The scale bar is 100 *μm*. **(B)** Bidimensional reproduction of the experimental cell environment, the hydrogel and chemotactic factor. This description is used as a base for our simulations. The *C*_0_ environment is defined as the sum of a linear gradient of a chemotactic factor distribution (*ν*_*c*_), increasing from green to yellow, and the extracellular matrix profile (*ν*_*ECM*_), in purple.

ECs tend to reach a quiescent mode forming an endothelium. This endothelium is formed at the border side of the hydrogel, mimicking the ECM in the side channel. We see a picture of the artificial ECM in (Figure 2A). Cells remodel and advance through the pores of the ECM, physically enlarging the pores and chemically degrading the extracellular matrix. Because of the changes cells produce in the ECM matrix liberating proteins, ECM-cell interactions become more intricate and relevant. The ECM conditions the behaviour of cells and acts as a scaffold to stabilize the structure of the forming vasculature network.

Angiogenesis has been shown to rely on a migratory “tip”-cell. A tip-cell guides migration and is followed by proliferative stalk-cells that elongate the sprout Carmeliet et al. (2009); Jakobsson et al. (2010). The most sensitive EC to the chemoattractant stimuli becomes a tip cell that guides the vessel sprout at the forefront. This tip cell forms numerous filopodia that scan the environment for angiogenic cues De Smet et al. (2009); Mayor and Etienne-Manneville (2016). We also see this collective behaviour in our experiments (Figure 1C). In this image we observe a collective of cells advancing a central cytoplasmatic finger shape that should become a capillary in time. The growth and dynamic evolution of this angiogenic sprouting is what we model and characterize in this study.

To study the behaviour of cells, as a collection, seen in (Figure 1C) we consider both the ECM structure and the chemotactic factor gradient present in our experiment. We model the chemotactic collective migrating phenomena conditioned by the ECM structure. First, we neglect the remodelling of the ECM through mechanical and chemical interactions as the investigation focuses on the response of cells to the presence of chemical gradients and ECM distribution; we assume the cell environment to be spatial and temporally static. Then, we proceed with a model extension to allow cells to remodel the cell environment by sensing it and degrading the ECM. Various computational approaches have been applied to cell migration, and angiogenesis Alert and Trepat (2020); Heck et al. (2015), but cell motility is an outstanding example of a moving boundary problem. For this reason, some authors have proposed a phase-field theory to model angiogenesis Travasso et al. (2011); Moure and Gomez (2021); Lima et al. (2014); Vilanova et al. (2017); Xu et al. (2017); Giverso and Ciarletta (2016); Santos-Oliveira et al. (2015). Phase-field methodology solves most of the challenges related to the numerical solution of moving boundary problems and is one current methodology that has shown promising results in modelling single and collective cell behaviour Santra et al. (2020). The dynamics of cells in the phase-field methodology are governed by a partial differential equation that minimises an energy functional at a low computational expense.

There are two main ways to model cells: continuum models, which treat cells as concentrations of cell types, and discrete models, such as agent-based or particle models, which consider individual cells.

Cellular Automaton (CAs), Cellular Potts Models (CPMs) and Phase-Field Models are all lattice-based models representing the system as a set of cells or a continuum. On the other hand, Agent-Based Models (ABMs) are not necessarily lattice-based and can be either discrete or continuum. ABMs and CAs both use a set of rules to update the state of the system. In CAs, the state of each cell is updated depending on the neighbouring cells; meanwhile, the behaviour of ABMs is determined by the collective interaction of each individual agent. On the other hand, CPM and Phase-Field models are driven by energy minimization. In CPM, cells are treated as discrete entities, a collection of mobile lattice sites; meanwhile, Phase Field Models represent a continuous field that gradually changes across a transition region. Phase Field models represent the interface between two materials or regions by a transition zone where the properties of the materials gradually change.

The agent-based and discrete models described models above, provide a good intuition of the processes involved in the cellular tip extension and migration but need to explore the influence of the surrounding environment, which scale is considerably larger. The main problem in using these discrete rules to capture the response of global systems with variable conditions and high interactivity is that they rely on something other than physical principles but statistical or heuristic rules that control the extension and branching phenomena. These limitations of discrete models regarding the underlying development mechanics are addressed with continuum modelling by using partial differential equations (PDEs) governing more global descriptions of the presence of endothelial cells. Therefore, in recent studies hybrid models, combining both approaches, have gained traction as it allow us to gain a more comprehensive understanding of the system. However, appropriate linking and calibration of such models should be performed, and parameters are only sometimes measurable.

CPM has been extensively used to study cell migration and proliferation in angiogenesis Graner and Glazier (1992); Szabó and Merks (2013) and has also been extended as a hybrid model to account for different cases such as cell heterogeneity and its impact on tumours Leschiera et al. (2022); Zhang et al. (2023) and mechanical cell-matrix interactions van Oers et al. (2014). Other hybrid ABMs and CA models have been proposed to study the interaction between tumour and immune cells Ghaffarizadeh et al. (2018); Norton et al. (2019); Hendrata and Sudiono (2019). A different approach, closer to the approach we have applied to this study, is the example of the model presented in Travasso et al. (2011), which is a hybrid model that combines an agent-based rule to simulate the behaviour of individual cells and a PDE to account for the continuous model to capture the evolution of capillaries and angiogenic factors. This same model has been extended and applied to numerous situations since it allows tailoring the vascular network and proliferation rate by adjusting its agent-based parameters. Vilanova et al. (2013) proposed a high-order phase-field approach using isogeometric analysis. This model was subsequently refined and extended by Xu et al. (2016, 2017); Vilanova et al. (2017); Xu et al. (2020); Moure and Gomez (2021). These studies demonstrate the potential of phase-field modelling to capture the complexity of intermediate-scale growth dynamics.

There is a gap when looking for formulations that combine the continuous description of intermediate-scale physics with continuous integration of the micro-environment components. This paper proposes a new approach integrating environmental chemical interactions with cells using the phase-field modelling spirit. Therefore, it can capture the dynamics at the represented level without using explicit or discrete strategies.

Here we elucidate the influence of the ECM based on phenomenological phase-field modelling. Through quantitative matching of experiment and theory, we first reveal the occurrence of cell migration driven by the distribution of chemotactic factors and the structural configuration of the ECM. In a self-consistent manner, such revelation reflects the coupling strengths of the constituent materials of the ECM and biological factors, allowing for their quantification in the model. As opposed to other models, we write a single equation to describe cell migration and couple the cell environment contribution into a single parameter, making it more straightforward and interpretable. Our study thus demonstrates an approach to developing a comprehensive and predictive understanding of physical coupling phenomena for a potentially broad family of ECM and the distribution of cells. In this model, we focus on the dynamics and evolution of the conformation of the experimental results shown in Figure 1C, considering the chemotactic factor and the hydrogel structure. We aimed to recreate the process of sprouting angiogenesis, which specifically involves the migration of specialised ECs. Our phase-field methodology, describing collections of cells, allows us to represent the cellular response to its environment, i.e., the ECM and the distribution of the chemotactic factors.

### Experiments

We perform the experiments using the angiogenesis platform design represented in (Figure 1A), which has been inspired by the microfluidic platform used in previously published experiments López-Canosa et al. (2021, 2022). Deposits on each side of the channel have different concentrations of chemotactic factors, generating a gradient across the ECM. We use the invasion of ECs into the central chamber, filled with a large-pore, low-concentration fibrin-based hydrogel with a mean pore size of 40*μm*, to study chemotactic cell migration. The microfluidic chip is designed to separate the different phenomena to isolate and investigate the behaviour of endothelial cells in response to chemical signals.

Details of the setup were published previously López-Canosa et al. (2022) and are further elaborated in the *“Microfluidic assay and cell culture preparation”* section of the Supplementary Material. Briefly, the hydrogel (fibrinogen and bovine thrombin mixture) is injected into the central chamber and left for cross-linking. Rat endothelial progenitor cells (rEPC) are introduced into one of the side channels of the chip. Then the chip is incubated for 45 min in an upright position, laying on the opposite side of the cells, to allow cell attachment to the central chamber. Finally, the reservoirs were filled with medium with 120 *μL* upstream and 90 *μL* downstream.

In these experiments, we use VEGF as a chemotactic factor, not only because it is a very well-known chemoattractant but also because angiogenesis is usually promoted by the expression and generation of a VEGF gradient that activates ECs Shibuya (2001). In our experiments, the VEGF gradient is generated solely by the diffusion of the molecules through the extracellular matrix López-Canosa et al. (2022).

After culturing the cells for 24 h, we introduce the chemotactic factor into the system to induce cell migration through the ECM. The reservoirs are refilled with medium and medium + VEGF every 24 h to keep the hydrostatic pressure difference between the reservoirs, which generates the flow in the side channels and allows us to have a constant gradient distribution as evidenced in López-Canosa et al. (2022), we see that the gradient of a 40 kDa dextran (which has a similar diffusion coefficient to VEGF) in the chamber is approximately constant after 15 h of diffusion in the central chamber of the experiment.

In this study, we develop a mathematical model based on the *in vitro* experimental results of cell migration in response to VEGF concentrations after 48 h. The endothelial cells were cultured for 24 h to enable endothelium formation before introducing VEGF into reservoirs on the other side of the central chamber, thereby creating an angiogenic factor gradient. Upon stimulation with an angiogenic factor gradient (0.1 *μg*/*mL*), a migration distance of 290 ± 53 *μm* was observed. This finding was derived in three separate microfluidic chips, each subjected to four measurements, resulting in a total of 12 migratory measurements. The experimental images can be found in Supplementary Figure S4 in the *“Microfluidic assay and cell culture preparation”* section of the Supplementary Material. In contrast, when no VEGF gradient was applied (0.0 *μg*/*mL*), the total cell migration was substantially lower, with a migration distance of 45 ± 26 *μm*. It is worth noting that the mean pore size of our hydrogel is 40 *μm*.The substantial increase in cell migration observed when the VEGF concentration was raised from 0.0 to 0.1 *μg*/*mL* highlights the profound impact of the VEGF gradient on endothelial cell migration and underscores the importance of VEGF in promoting angiogenesis. Based on these findings, our experiment indicates that stimulating cells with chemotactic factors is essential for effective migration. In the absence of a chemotactic factor gradient in the central chamber, cells do not migrate through the hydrogel but instead, fill the nearest pore.

In this study, the model focuses solely on a single cellular lineage, specifically endothelial cells, and does not account for other cell types, such as pericytes, which play a crucial role in vessel stabilisation and maturation. This simplification limits the applicability of the model to more complex *in vivo* situations. Second, the model only considers chemotaxis as the driving force behind cell migration without incorporating other forms of cell guidance, such as durotaxis or haptotaxis. The exclusion of these additional mechanisms may result in an incomplete understanding of the various factors contributing to cell migration *in vivo*.

The experiments conducted in this study aim to validate and compare results with those derived from our newly developed mathematical model. The chosen experimental conditions and mathematical model were specifically tailored to the system designed: we utilised a low concentration of fibrinogen, yielding a soft hydrogel with large pores conducive to cell size. It is important to note that this experimental setup represents only one aspect of the broader potential biological conditions, as it is intended to isolate the different migration contributors. In fact, we acknowledge that more complex, in vivo-like conditions, including varying sizes of pores, macropores, micropores, and different structures, might significantly impact cell migration patterns and angiogenesis.

## Model

This work proposes a dynamic phase-field model to describe the process of sprouting angiogenesis, where a collection of cells advances through a porous matrix responding to chemotactic factors. The model is based on a modified Cahn-Hilliard equation that incorporates the effects of the local environment on the angiogenic sprout growth. We are able to write in a single equation the whole migration model by only considering an extra contribution for the environmental effect without using agent-based terms. The governing equation is as follows:

$$\frac{\partial \phi }{\partial t}=M\nabla^{2}\left(\underset{\text{Cahn}-\text{Hilliard}}{\underset{}{-\phi +\phi^{3}-\epsilon^{2}\nabla^{2}\phi }}+\underset{\text{Environment effect}}{\underset{}{2\phi \epsilon C_{0}B_{\phi }-\epsilon^{2}\nabla^{2}B_{\phi }}}\right).$$ (1)

Our formulation uses an order parameter, *ϕ*, to describe the presence or lack of cells in two phases taking values ±1 at the bulk. Here, *ϕ* represents the phase field variable characterising the angiogenic sprout interface, with *ϕ* = 1 denoting the sprout and *ϕ* = −1 indicating the surrounding environment. The term $\frac{\partial \phi }{\partial t}$ signifies the rate of change of *ϕ* concerning time. *M* represents mobility, a proportionality constant that determines the speed of interface evolution. It can be thought of as a proportionality constant that relates the rate of change of the phase field variable to the driving forces of the system. The value of *ϵ* controls the width of the diffusive interface connecting the two phases, with a hyperbolic tangent profile, where *ϕ* values are close to zero. The first term of Eq. 1, the Cahn-Hilliard term, derives from a Ginzburg–Landau free energy written in dimensionless form Lacasta et al. (1993). The Cahn-Hilliard term, −*ϕ* + *ϕ* ^3^ − *ϵ* ^2^∇^2^ *ϕ*, is responsible for the energy minimisation of the system. The terms −*ϕ* and *ϕ* ^3^ respectively favor a homogeneous distribution and phase separation between the sprout and its environment. The gradient energy term, *ϵ* ^2^∇^2^ *ϕ*, penalizes large gradients of the phase field variable, promoting smooth interfaces. The environment effect term, 2*ϕϵC*_0_ *B*_*ϕ*_ − *ϵ* ^2^∇^2^ *B*_*ϕ*_, accounts for the influence of the local environment on angiogenic sprout growth. *B*_*ϕ*_ denotes the biochemical signal guiding the sprouting process. The term 2*ϕϵC*_0_ *B*_*ϕ*_ represents the coupling between the phase field variable and the biochemical signal, promoting or inhibiting sprout growth depending on the local concentration of *B*_*ϕ*_. The term −*ϵ* ^2^∇^2^ *B*_*ϕ*_ indicates the effect of the gradient of the biochemical signal on sprout growth, favouring growth in the direction of increasing *B*_*ϕ*_. The total angiogenic factor, *C*_0_, represents the environment in our model which incorporates the porous media and chemotactic factors, illustrated in Figure 2B), defined by

$$C_{0}\left(x,y,t\right)=\nu_{c}\left(x,y,t\right)+\nu_{\mathrm{ECM}}\left(x,y,t\right).$$ (2)

We assume that the dynamics of cells as a whole collection result from the balance between the biological agents present in their environment: the distribution of chemotactic factors (*ν*_*C*_) and the presence of the extracellular matrix (*ν*_*ECM*_). This reduced parameterisation improves the interpretability of the model by grouping the effects of the chemoattractants and the ECM. It is important to note that while the ECM can support cell migration by providing a physical framework, it can also hinder migration by creating barriers and obstacles for cells to overcome. Including the chemoattractants and the ECM in the model can capture this complexity and provide a more accurate representation of cell migration dynamics.

*C*_0_ represents three different environmental situations in our system: hydrogel alone, hydrogel combined with chemotactic factors and pore with chemotactic factors. In this article, if the contrary is not said, *ν*_*c*_ is taken as a linear gradient distribution (*ν*_*c*_(*y*) = *αy* + *β*) and *ν*_*ECM*_ is based on the microscopy image of the hydrogel presented in (Figure 2A). Cells advance naturally if *C*_0_ is positive, but if it is zero or close to zero, cells do not feel its effect and remain immobile, allowing us to define the different situations cells encounter in the environment, (Figure 2B), quantitatively. The dark purple zones represent the densest ECM, or hydrogel, regions $\left(C_{0}\left(\nu_{\mathrm{ECM}}\right)<0.1\right)$ , which do not promote cell advancement and only favours the presence of cells if positive. The green to yellow background represents the VEGF increasing concentration, *ν*_*c*_ that activates cell migration $\left(0.2<C_{0}\left(\nu_{c}\right)<0.5\right)$ . There is also a very narrow interface between the hydrogel structure and the pore with chemotactic factor concentration $\left(0.1<C_{0}\left(\nu_{c}\right)<0.2\right)$ that promotes cell attachment to the surface of the hydrogel.

Following the description of Eq. 1, *B*_*ϕ*_ is the interaction term describing the influence of the cellular environment on cells. Tip cells lead the migration to proangiogenic and chemotactic cues present in their microenvironment Siekmann et al. (2013); Isogai et al. (2003); Gerhardt et al. (2003). Therefore, accounting for the minimal proliferation rate of tip cells Siemerink et al. (2012); Yetkin-Arik et al. (2019) and the time scale of our model, we have dismissed proliferation and allowed cells to migrate from the endothelium, modelled as a reservoir by using Dirichlet boundary conditions. *B*_*ϕ*_, which communicates the effect of the microenvironment on the model, reads as:

$$B_{\phi }=\epsilon C_{0}\left(\phi^{2}-1\right).$$ (3)

The first term of the environment effect, 2*ϕϵC*_0_ *B*_*ϕ*_, is derived from a chemotactic potential that drives and nurtures cells. The second term of the environment effect, *ϵ* ^2^∇^2^ *B*_*ϕ*_, is a diffusive driving force that migrates cells at the cell interface. *B*_*ϕ*_ is proportional to *C*_0_ and centred at the limit of cells, keeping its effect far from the bulk cells, based on the rich cell-surface receptors and numerous filopodia present on tip cells. In our model, endothelial tip cells appear naturally by the localized impact of the biological factors at the interface; the non-homogeneity of the microenvironment promotes the advancement of cells with no additional rules. The values of the parameters used in the simulations are detailed in Table 1.

**TABLE 1:** Numerical integration parameters of the phase-field and physical equivalencies.

Symbol	Description	*In Silico* value	Physical value
L	System size	200	770 *μm*
Δ*x*	Spatial discretization size	1	3.85 *μm*
Δ*t*	Temporal discretization size	10^–3^	2.75 ⋅ 10^−6^ *h*
*ϵ*	Phase-field interfacial length scale	1	3.85 *μm*
*M*	Phase-field mobility coefficient	1	$5.11\cdot 10^{3}\frac{1}{h}$
$C_{0}^{\min}$	Modelisation of hydrogel	0 ≤ *C*_0_ ≤ 0.1	Hydrogel
$C_{0}^{\mathrm{int}}$	Modelisation of hydrogel-pore interface	0.1 ≤ *C*_0_ ≤ 0.2	Hydrogel with chemotactic factors
$C_{0}^{\max}$	Modelisation of chemotactic factors	0.2 ≤ *C*_0_ ≤ 0.5	Pore with chemotactic factors
*αy* + *β*	Linear chemotactic factor gradient	0.1*y* + 0.3	$\frac{100ng-0ng}{1mm\cdot mL}$

Our model conserves the number of cells in the system by using a conserved phase-field. Therefore, the initial condition determines the number of cells in the system and the number of mobile cells. However, to enable the advancement of cells as a collection, we consider the endothelium at the side-channel of the microfluidic platform acting as a reservoir of cells that injects cells into the system if necessary; cells stimulated chemotactically for advancement, *C*_0_ > 0 naturally come out from the reservoir. Dirichlet boundary conditions model this reservoir. To compute this migration flux, we use the total volume of the system at a given time in terms of its scalar field *ϕ* and divide it by the time:

$$\frac{\Delta V\left(t\left(x\right)\right)}{\Delta t}=\frac{\sum_{\forall i,j}\phi_{i,j}\left(t\left(x\right)\right)-\sum_{\forall l,k}\phi_{l,k}\left(t_{0}\left(x\right)\right)}{t-t_{0}}.$$ (4)

Because the migration of ECs is oriented and regulated mechanically and chemically, we assume that the tip cell directs migration towards a clearer path. Moreover, EC collective migration also involves the degradation of the ECM to enable the progression of cells Lamalice et al. (2007); Yana et al. (2007); Koziol et al. (2012); Minor and Coulombe (2020); Song et al. (2018). For this reason, we extended the model by introducing a rule for tip ECs, allowing them to also degrade the ECM by producing matrix metalloproteinases (MMPs) and generating proangiogenic factors Seiki (2002); Haas and Madri (1999).

Several limitations are intrinsic to the mathematical model. Currently, the mathematical model is implemented in a two-dimensional (2D) framework, which, while simplifying the computational problem, has inherent limitations in its ability to represent the full complexity of 3D EC behaviours. We further assumed that the hydrogel is a homogeneous and isotropic material which does not accurately reflect the actual physical and mechanical properties of the fibrin-based hydrogel. Furthermore, the model considers only one type of cell. The interactions between endothelial cells and the hydrogel are simplified as chemoattractant gradients, neglecting mechanical interactions or adhesion between cells and the hydrogel. Lastly, the model is limited to a two-dimensional system and treats endothelial cells as a continuous phase without explicitly modelling individual cells, which may not accurately represent the three-dimensional complexity of the cellular microenvironment *in vivo*.

### Degradation

**FIGURE 3:**
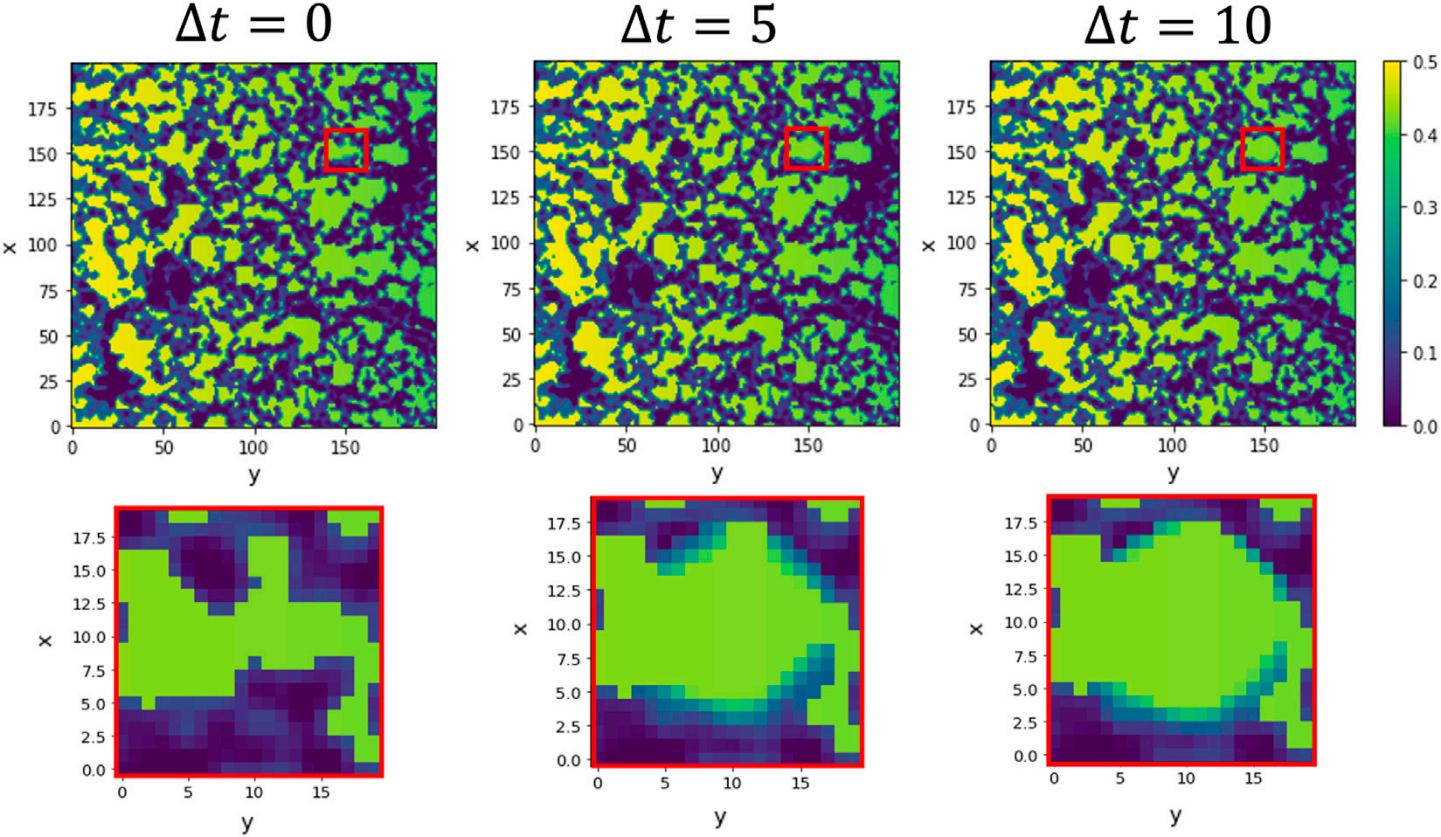
Simulation of hydrogel testing and degradation for a static tip cell. The simulation shows snapshots of the ECM (purple) and chemotactic factor (green) at the tip sensing area (red square) for an immobile tip cell. The tip cell can sense approximately two cell distance (10-pixel distance) to encounter the path of lower ECM following the increasing chemotactic factor. It liberates matrix-degradation enzymes such as matrix metalloproteinases (MMPs), modelled in a Gaussian-like distribution, to increase the pore size. The first row shows the whole ECM domain, and the bottom row shows the tip cell sensing environment and its surrounding evolution when, without advancing, the tip cell produces MMPs. We show 0, 5 and 10 time steps from left to right for a 0.2 degradative power.

The ECM is a physical object directing, promoting and hindering cell motility depending on its physical and mechanical properties (porosity, pore size, rigidity, etc.) Zhang Y. et al. (2021); Cao et al. (2021); Bružauskaitė et al. (2016); Narayan and Venkatraman (2008); Qazi et al. (2019). Studies indicated that endothelial cells could sense patterns of extracellular distributions and interpret gradient directionality in 3D collagen gel for distances of the order of 100 *μm* Korff and Augustin (1999); Gerhardt et al. (2003). Moreover, the fact that the production of MMPs influences EC migration has been known for a long time. Therefore, based on the experimental observations, we also include in this model the ability of tip ECs to sense their environment and degrade the extracellular matrix through MMPs Hosseini et al. (2015); Gálvez et al. (2001). An example of how this mechanism is implemented is shown in Figure 3. Because of the bi-directional cell-ECM interaction, during the simulations of the extended model with degradation, the ECM is remodelled and re-biosynthesised as the tip ECs migrate Davis and Senger (2005). We assume that tip cells can sense the microenvironment approximately at a two-cell distance, approximately 40 *μm* from its tip, to direct migration. We base this behaviour on the combination of the multiple cell mechanisms combined to help acquire directionality and guide the cell pathfinding in response to the ECM cues (focal adhesions, filopodia, lamellipodia, dactylopodia, tip endothelial cells surface receptors and stalk cells receptors) Fischer et al. (2019); Figueiredo et al. (2021); Phng et al. (2013); Smet et al. (2009). Our model allow activated tip cells to avoid highly dense ECM regions and migrate through the ECM, taking advantage of the pores. More information about implementing this mechanism into the model can be found in the Supplementary Material.

### Numerical integration

The implementation of our model is based on a 2D framework. This decision was made for simplification and computational efficiency, aiming to shed light on the complex dynamics of sprouting ECs. However, we emphasise that our 2D-implemented model inherently possesses limitations. Although it has provided valuable insights, the translation of these results to a more physiologically realistic three-dimensional (3D) environment should be approached with caution. In the 2D implemented model, cell behaviours and cell-cell and cell-matrix interactions might be simplified compared to those in a 3D setting. For example, cell migration patterns, as well as the distribution and perception of chemotactic gradients, could differ significantly between 2D and 3D contexts. Our ongoing research involves extending this model into the third dimension to capture more realistic cellular behaviours and interactions in a 3D environment.

**FIGURE 4:**
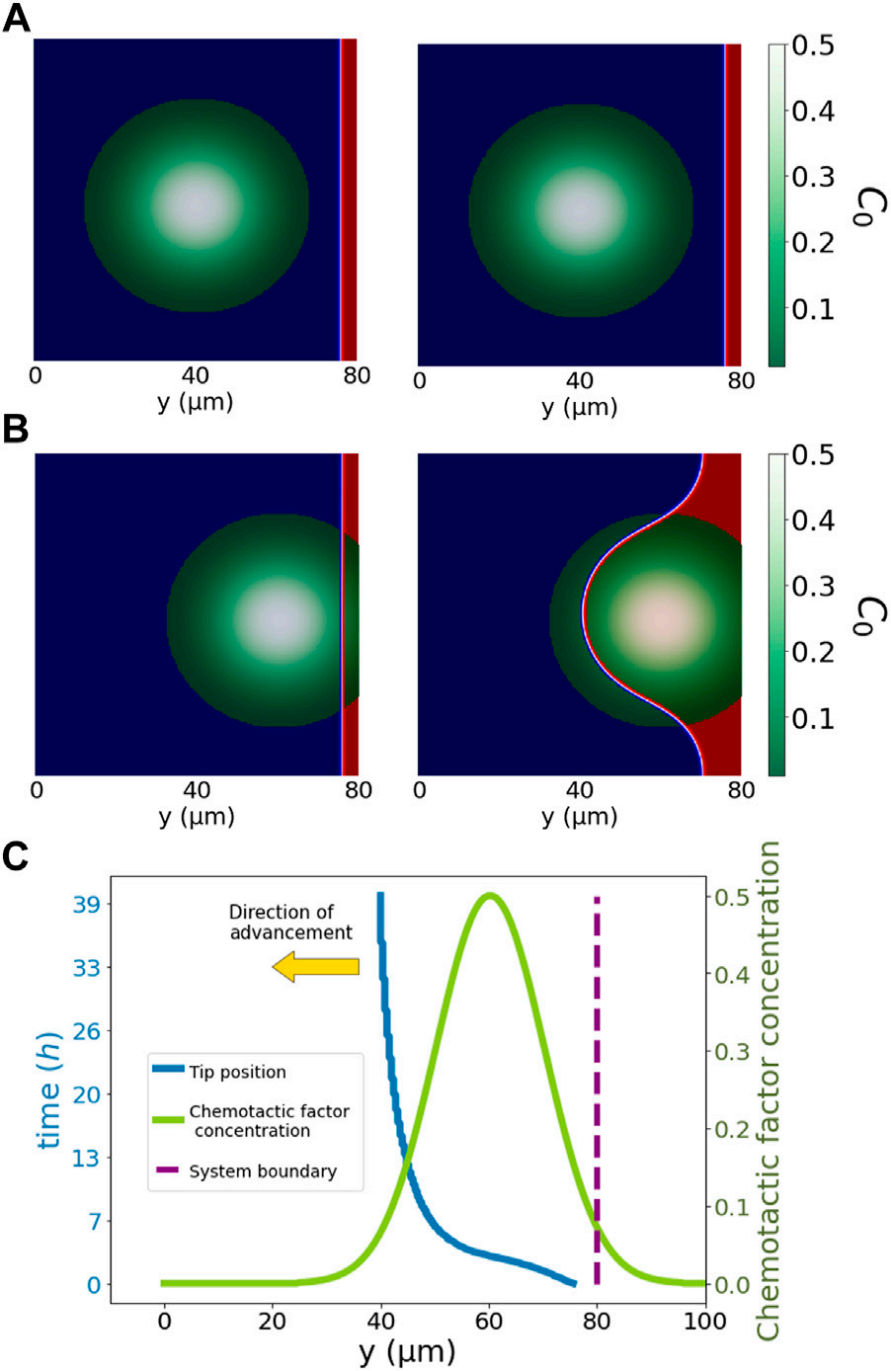
Simplified pore system. The simulation in the top row, **(A)**, shows snapshots of the cells (red) far from the pore and chemotactic factor concentration (green to white). The middle row, **(B)**, shows the results of an analogous simulation in which the pore and biological factors are in contact with cells. Cells, *ϕ* = 1, advance from right to left, i.e., from *y* = 200 towards *y* =0. For **(A,B)**, we have the initial condition on the left, and on the right, we have the system at time *t* = 27.5*h*. Cells advance towards the maximum chemotactic factor concentration, i.e., from *y* = 200 towards *y* = 0. Red represents the position of cells (*ϕ* = +1) and blue (*ϕ* = −1) the non-cellular region. The bottom row, **(C)**, illustrates the position evolution of the tip of the cells in **(B)** at the centre of the vertical axis, i.e., *x* = 100. Cells respond only to local biological factors, advancing towards the maximum chemotactic factor concentration. The advancement of cells is fastest at initial times with a clear direction of the gradient of chemotactic concentration. In later stages, the velocity of advancement reduces, and the tip expands, occupying the pore region. Displayed in green is the chemotactic concentration distribution, and in blue is the tip position, both sharing the same *x*-axis *y*. We displayed the simulation time for the tip cell position on the left vertical axis. In the right vertical axis, we displayed the chemotactic factor concentration. The yellow arrow indicates the direction of advancement.

**FIGURE 5:**
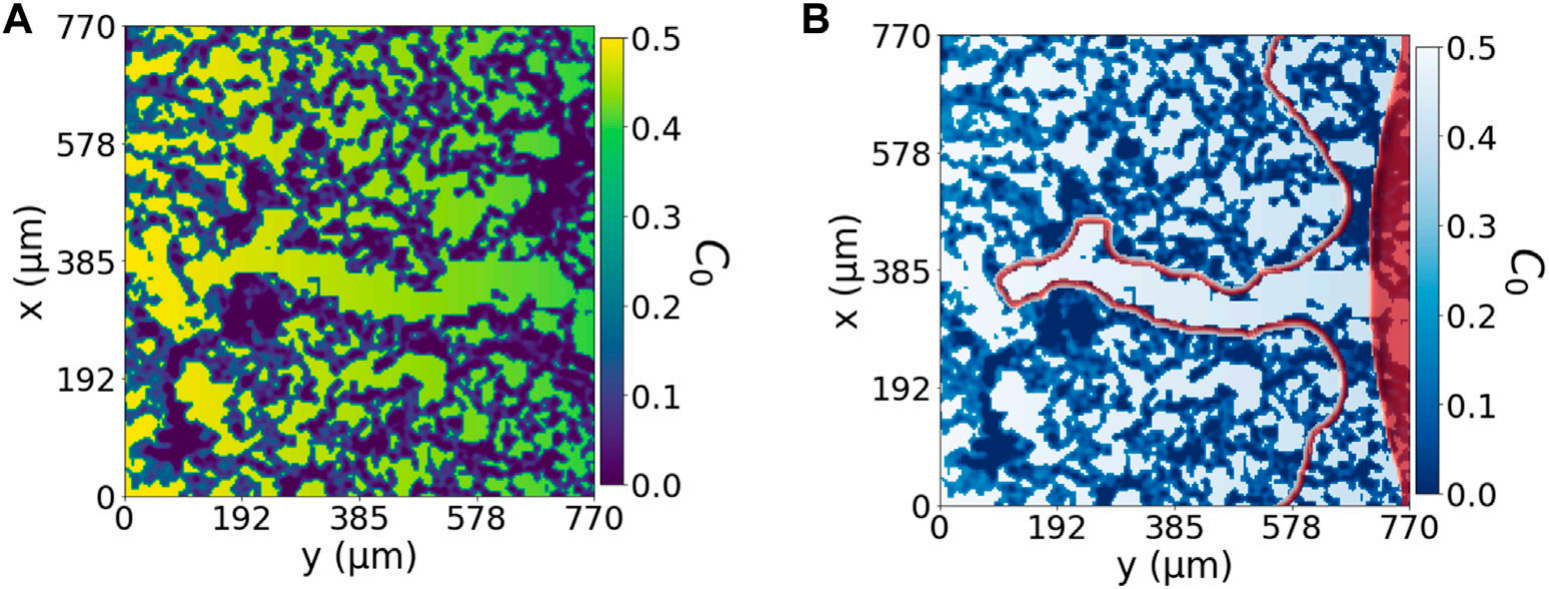
Fundamental dynamic model with lateral reservoir. **(A)** Represents the domain of the problem based on Figure 2) with an open path. *C*_0_ includes the effect of the extracellular matrix and the linear gradient of chemoattractant factors, increasing from right to left. **(B)** Cell advancement through the *C*_0_-landscape shown in panel **(A)**, from the *C*_0_ minimum on the right to the *C*_0_ maximum on the left. The red area represents the initial condition of a spherical endothelium, and the red perimeter represents the enclosing cellular region at simulation time *t* = 96.25 h.

**FIGURE 6:**
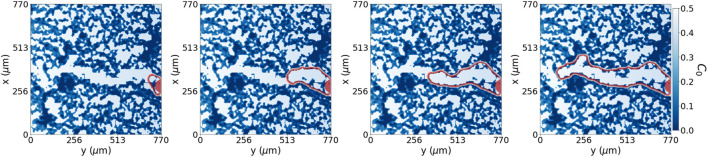
Fundamental dynamic model. Collective cell migration, with a small spherical reservoir and initial condition (filled in red), through an open path with no extracellular matrix degradation at *in silico* times {2.75, 27.75, 55, 96.25} hours, respectively from left to right. In red is the perimeter of cells advancing through the system.

**FIGURE 7:**
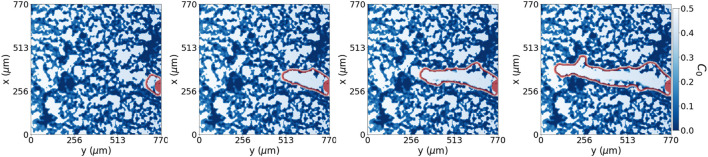
Dynamic model with degradation. Collective cell migration with extracellular matrix degradation at time steps {2.75, 27.75, 55, 82.5} hours, respectively from left to right. We see the perimeter of cells in red advancing through the system. The collective cell migration can sense the densest regions of the extracellular matrix and avoid them while breaking down and migrating through lower-density extracellular matrix regions in the system.

In all simulations, reservoirs of the two phases have been placed by imposing Dirichlet boundary conditions. The reservoir of cells (*ϕ* = +1) has been placed at the boundary of the system for the simulations are shown in Figures 4, 5 and with the same distribution as the initial condition for simulations shown in Figures 6, 7.

To scale our *in silico* system to the represented physical system, we used the actual measures of the fluorescence microscopy image of the fibrin-based hydrogel. The hydrogel image has a height and width of 775*μm*, and we converted it into a 200*x*200 pixel image, giving a pixel length of 3.875 *μm*, which we truncated to 3.85 *μm*. Therefore our system size is *L* = 200 = 770 *μm*. The unit of time *in silico* is considered to be *t* = 3.0 ⋅ 10^−3^ *h*. All the rest of the parameters have been converted accordingly. *ϵ*, a parameter controlling the interface of our phase-field system, is set to be small compared to the size of the system (*ϵ* = 1 ≪ *L*) for proper convergence Rogers et al. (1988). If not stated otherwise, the Table 1 show all the parameter values used in our simulations. The continuum model is implemented in a vectorial form using Python and solved using the five-point stencil for spatial finite differences and an Euler scheme for the time dependence.We summarise in Table 1 the dimensionless values of the parameters used in the model and the corresponding physical values obtained using the size of system *L* = 770 *μm* and the total duration of the experiment, *T* = 48*h*. The mobility of the phase field that defines the location and advancement of endothelial cells is set by the timescale of the dynamics of capillaries, defined through Δ*t*.

## Results

(Figure 1A) depicts our experimental arrangement. As demonstrated in (Figure 1C), within our experimental system, the protrusive structures of migrating cells stretch to 290 ± 53 *μm* upon stimulation with a VEGF gradient of 0.1 *μg*/*mL* over 2 days. In contrast, when the system was not stimulated with an angiogenic factor gradient (0.0 *μg*/*mL*), cell migration was significantly reduced to a distance of 45 ± 26 *μm*. This sustained migration pattern gives rise to the capillary structures observed during angiogenesis. This pattern closely emulates natural capillaries and effectively demonstrates the characteristics of early-stage angiogenesis. In our in-silico model, we incorporated these experimental findings and developed a mathematical model to simulate cell migration in response to VEGF gradients. The model successfully replicated the experimentally observed migration distances under both conditions. In our migratory assays, there is some variation in cell migration through the extracellular matrix (ECM), as shown in (Figure 2A). This variation in collective cell migration can be attributed to the random structural differences present across various regions of the ECM, a factor that significantly influences cell migration.

To work with our model, we begin with a digitalised description of the cell migration results shown in (Figure 1C) by gray scaling and normalising the image and selecting a colourmap to highlight the presence of the cytoskeleton. Figures 1B, C) show cytoskeleton distribution for cells after 48 h without VEGF and under the influence of VEGF concentration gradient in our microfluidic assay. (Figure 1C) shows random small features indicating partial penetration and exploration of the cells into the ECM.

To gain mechanistic insights into various global and local behaviour of EC migration in our system, we have performed phase-field modelling. Here, we concentrate on the coupled influence of the ECM and the effect of VEGF, chemotactic agents, in cell migration. The ECM, by definition, is the surrounding material providing structural and biochemical support. The environment of cells in our phase-field model is comprised of two components: the hydrogel structure and the angiogenic factor distribution, *ν*_*ECM*_ and *ν*_*C*_, respectively. In the following, we explore the advancement of cells reproduced by coupling these components into the *C*_0_ coefficient. We discover the apparent modulation effects of the ECM structure on the final cell morphology. Further, we allow cells to sense and degrade the ECM structure to reproduce cell migration.

Figure 4 presents a simplified system for cell advancement in a pore filled with chemotactic factors in the modelled ECM. In the first row, sprouting angiogenesis is not triggered due to the spatial distance between cells and the biological factors (the pore and the chemotactic factor). By contrast, panel B) in the second row shows a system where the pore filled up with chemotactic factor is in contact with cells, triggering invasion of cells into the pore region. Cells advance only with the local presence of positive values of total factor concentration, *C*_0_ (chemotactic factor and no dense ECM). The overall migration depends on the total angiogenic factor distribution (*C*_0_) and the initial morphology of cells. Our model captures all essential attributes of cellular migration observed in our microfluidic assay, including pore and ECM invasion and chemotactic response. (Figure 4C) depicts the tip cell position (most advanced position *y*-direction wise) across the system and the *C*_0_ distribution as the chemotactic factor increases from 0.1, on the initial position of cells, to 0.5 and then decays to 0 again. Cells gradually invade the pore to follow the gradient direction of the angiogenic factor distribution. After the chemotactic distribution peak, the gradient direction disappears, resulting in a more isotropic cell advancement. The cell velocity varies with the change of *C*_0_ value and distribution. The velocity of cells is high until they reach the centre of the distribution, where their migration morphology changes from a sprouting penetrating tip towards an area-widening invasion to fill up the pore area. In this second regime, the advancing tip velocity substantially decreases because migrating cells no longer move towards the *y*-direction but homogeneously to the empty pore. A further decrease in cell migration velocity happens when the *C*_0_ value decreases below 0.1. The drop in cell velocity below a specific value indicates the lower bound of the total angiogenic factor, representing the interaction between cells and the ECM. Besides, the shape modulation of the ECM also causes cellular morphology adaptation, presumably through mechanical interactions of the actual system.

Figure 5 shows a numerical example of a representation of a domain of 750 *μm* by 750 *μm* of the central chamber in the experimental system. It has the same configuration as shown in (Figure 2B): a hydrogel structure with a general chemotactic factor gradient. In this case, the environment is static; cells are not able to modify it. For this reason, we have constructed a biomimetic pore in the ECM which cells follow. If this path was not created, the endothelium under the influence of this same environment as in Figure 2B), advances as a whole layer, activated by *ν*_*c*_, following the chemotactic factor gradient. Activated migratory cells, unable to change their environment, sense the effect of the hydrogel generating small protrusions according to the ECM pores and irregularities. However, these protrusions are quickly absorbed by the advancing front of cells. However, by placing an open path, cells generate an invading protrusion that advances faster than those outside the pore that encounter the irregular ECM. Figure 5 shows that migrating cells adapt their shape to the morphology determined by the hydrogel while advancing towards the increasing chemotactic gradient. This result can be directly compared to Figure 1C, where cells migrated collectively towards a concentration gradient. Looking at the top and bottom, *x* = 0, 200, of Figure 5, we can also see that cells that do not encounter a clear open path also advance due to the chemotactic factor activation but at a much slower pace because of the hindering of the ECM.

This system, Figure 5, also explicitly shows how cells can advance over the irregular hydrogel pores slower than through the open trail. This tip cell advancement is comparable not only to Figure 1D but also to the results obtained by Wang et al. (2021) where ECM-cell interactions regulate the sprout diameter. We illustrate the dynamics of our model in Figure 6, where we changed the initial condition to an initial sphere, which will also act as our cell reservoir, to solely focus on the tip advancement that is the region of interest in our model. We see that this system with a lower pool of cells as the initial condition advances at the same speed and gives the same morphology as Figure 5. This result is not surprising as the advancing mechanism’s driving force is effectively the pore’s chemotactic factor distribution. The unique difference is the volume change, given that in Figure 5 we have the lateral cues also growing.

### Dynamic model with degradation

The advancement of cells through the ECM depends on open pores and the remodelling of the surrounding. Cells placed in a porous material must open their path through the ECM to migrate by exerting forces onto the ECM and degrading it, producing MMPs. For this reason, we extend our model by allowing tip cells to sense their environment and degrade, or re-biosynthesise, the hydrogel, converting it into pro-migratory agents Naito (2000). For simplicity, we have assumed that the production of MMPs from the tip is enough to avoid advancement hindered due to the presence of ECM. Using the capability of tip cells to sense their surroundings, we place the MMPs distribution according to the hydrogel concentration region sensed by the tip. Therefore the tip cell degrades the ECM, avoiding highly concentrated hydrogel regions as seen in Figure 7.

We can represent multiple scenarios by tailoring the MMPs production and its centring variability concerning the tip position. It is important to note that the degradative power and MMPs production play an essential role in deciding the tip path.

In Table 2 we summarize our experimental and numerical results. Consistently with our experiments, our model for cell migration predicts tip velocities of the same order of those obtained in our experiments. Because the driving mechanism for migration, chemotaxis, has the same profile in both models, we have obtained very similar mean velocities (differences due to path tortuosity) and migration rates (phase volume difference over time ratio) for the fundamental model and the model with degradation. In this model, the migration speed can be tailored to specific cases by changing the background gradient and the initial conditions of the system.

**TABLE 2:** System results. The *fundamental dynamic model* refers to Figure 5.A2-B2) and the *Dynamic model with degradation* refers to Figure 7. For each figure, average velocities have been computed between the initial and final conditions. The negative sign of mean tip y-velocity appears from cells advancing from *y* =200 towards *y* =0. *V* (*t*(*y*)) corresponds to the volume of cells in the system when the tip is at *x* as in Eq. 4 but subtracting the initial volume state and time, and *px* refers to pixel.

	*In vitro* system	In silico systems	
	*Endothelial cells*	*Fundamental dynamic model*	*Dynamic model w. Degradation*
Domain size	375 *μm* × 250 *μm*	775 *μm* × 775 *μm*	775 *μm* × 775 *μm*
Average Pore size	40 *μm*	40 *μm*	40 *μm*
Chemotactic factor gradient	−0.1 *μg*/*mL*	$-3.85\cdot 10^{-3}\;\frac{\#}{\mu m}$	$-3.85\cdot 10^{-3}\;\frac{\#}{\mu m}$
Mean tip y-velocity	−6.1 *μm*/*h*	−6.2 *μm*/*h*	−7.4 *μm*/*h*
Pixel area	1.8*μm* × 1.25 *μm*	3.85*μm* × 3.85 *μm*	3.85*μm* × 3.85 *μm*
$\frac{V\left(t\left(y=25\right)\right)-V_{0}\left(t\left(y=180\right)\right)}{t\left(y=25\right)-t\left(y=180\right)}$		0.668 ⋅ 10^−3^ *h* ^−1^	0.774 ⋅ 10^−3^ *h* ^−1^

## Discussion

Our study does not consider the effects of fluid flow and shear stress on cell migration, as the design of our microfluidic setup aims to minimise these factors. However, it is important to recognise that under physiological conditions, both fluid flow and shear stress can significantly influence cell behaviour. Nonetheless, despite these constraints, our model offers crucial insights into the behaviour of endothelial cells in a controlled setting. These findings, in turn, can help inform future studies that may incorporate more nuanced and realistic microenvironment features.

To shed light on the fundamental interactions within an actual cell microenvironment, as well as to predict cell performance within a porous material, we developed a model grounded on microfluidic assays with rEPC, as detailed in Figure 1. The experimental model developed in this study applies to large-pore, low-concentration fibrin gels. It is here that the model’s robustness and applicability have been most effectively demonstrated. This platform enables us to isolate the effects of extracellular matrix (ECM) and the chemotactic factor, while effectively eliminating the influence of confounding parameters like fluid convection and flow Polacheck et al. (2011); Kingsmore et al. (2018).

The generation of capillaries is a complex process that highly depends on localised degradation of the ECM and subsequent EC migration, proliferation, and differentiation. ECs remodel the ECM during the invasion, and the ECM influences the shape and morphology of migratory cells. For ECs to form capillary-like structures, fibrin-based gels or ECM must be sufficiently soft to allow ECs to generate space to grow Kniazeva and Putnam (2009). The obtained results with our model elucidate the central role of the ECM during collective endothelial cell migration and organisation.

This work evaluated the capacity of the presented model to replicate the collective EC migration through a fibrin-based hydrogel. The hydrogel structure and composition, represented by *ν*_*ECM*_ in our model, are critical factors for the vascularisation process. In this work, we have focused on the structure of the hydrogel but have not compared or explored the adequacy of the specific ECM by changing the value of *ν*_*ECM*_ describing the ECM.

To introduce this new numerical model, we addressed the capability of chemotactic factors, such as VEGF, to promote cell migration in our experimental model. For this purpose, we tested our numerical system by testing the effect of a punctual diffusion source in a pore; see Figure 4. When ECs sense the chemotactic factor, they migrate towards the increasing gradient, agreeing with our experiments. On the other hand, for a homogeneous gradient, the cell advancement predicted in our model is also homogeneous, similar to epithelial sheet wound healing Nikolić et al. (2006). Introducing irregularities in this system, such as the ECM structure with no clear open paths or large pores, it is possible to further reproduce the small tips at the front of the epithelial sheet wound healing. If irregularities are not sustained during the advancement of cells, the advancing sheet of cells again absorbs the spontaneously formed tip structure. Therefore, to generate sprouting structures, we need a sustained inhomogeneity in our domain - which cells produce by degrading their surrounding. Similarly, we can use a punctual distribution of chemotactic factors or formed open paths through the ECM to describe sprouting-EC migration. Regions with irregular ECM hinder the advancement of ECs compared to open paths or pores, favouring fingering morphology led by the tip EC advancement as shown in Figures 5, 6. These finger-like shapes are a break of symmetry promoted by the surroundings. Therefore, bifurcations and multiple-fingering morphologies would naturally emerge given the appropriate surroundings with no need for extra rules conditioning the behaviour of the model.

The model with ECM degradation, shown in Figure 7, allows for dynamic matrix remodelling, an essential factor in angiogenesis. This process enables cells to form new pathways, leading to potentially more efficient and directed migration. The key distinction we observed relates to the tortuosity of the established pore, which impacts the *y*-axis tip velocity. By incorporating ECM degradation, we can see the effects of a variable and complex microenvironment on cell sprouting. However, the rate of degradation plays a crucial role. Overly rapid and extensive degradation could destroy the ECM, leading to unrealistic migration patterns. Conversely, a low degradation rate may restrict new pathway formation, thus limiting cell penetration. In contrast, the model without ECM degradation presumes a static ECM which takes advantage of the existing path. This limitation could lead to slower, less directed migration as cells are confined to the existing pore morphology.

The capability of cells to sense and migrate in their environment is a very complex process that influences cell migration. By allowing cells to sense and degrade their surrounding in our model with degradation, we can replicate the migratory behaviour seen in our experiments without manipulating the ECM structure, see Figure 7. Because we do not have more information in middle times, we set the degradation power of cells high enough not to hinder cell migration, as can be seen from the results shown in Table 2. At each time discretisation step, cells sense their surroundings, as in Figure 3, and decide to move accordingly to the path of lower environment resistance. We see how the average tip y-velocity in the case of ECM degradation is higher than the one with the pore already generated. The dynamic model with degradation opens a pore through the ECM more straight than the one defined for the dynamic model with no degradation. Besides, the phase field flux defined in Eq. 4 allows us to quantify the driving force stimulating the tip for a given set of initial conditions. The observed flux in both cases, with and without degradation, is of similar magnitude, albeit slightly higher in the case with ECM degradation. This outcome is anticipated since the chemotactic gradient profile remains the same for both scenarios. However, it is noteworthy that even a small portion of the ECM present in the path of migrating cells has the potential to contribute to subtle variations in cell sprouting behaviour and the establishment of gradients. This insight suggests that the degradation property and gradient profile can be readily adjusted to accommodate specific cases where the ECM may exhibit different tendencies toward degradation or remodelling.

While we have gleaned important insights into the dynamics of EC sprouting from our 2D implemented mathematical model, we recognise that these results have limitations when interpreted in a 3D physiological environment. For instance, cell behaviours and cell-cell and cell-matrix interactions in a 2D model can differ substantially from those in a more physiologically realistic 3D environment. A 3D environment might exhibit increased complexity in cell migration patterns due to the additional spatial degree of freedom, and the chemotactic gradients could distribute differently in a 3D context, influencing cell behaviour in ways not captured by our 2D implementation model.

We are actively working on extending our model into three dimensions to represent and predict EC behaviours in 3D experimental setups more accurately. Furthermore, we are employing additive manufacturing techniques together with different porogens to generate 3D structures with enhanced control and simulate a multitude of conditions with pores, in the micro and nanoscale. This will allow us in the future to compare the predictions of our mathematical model with cellular experiments under a wide range of conditions.

We have also confirmed that the dynamics or advancement of our model highly depends on the chemotactic factor distribution, which controls the flux of cells from the reservoir. Therefore, by scaling the chemotactic gradient and changing the cutoff of our ECM distribution, our model can reproduce multiple scenarios representing biomaterials with different morphologies. We believe that this model is an essential step toward modelling ECM-guided cell migration. Future developments and experiments should examine the effects of changes in the ECM on migrating cells and mechanical deformations on the same ECM structure.

## Conclusion

We proposed a phase-field model for cell migration that considers the ECM structure and chemotactic factors. From the mechanical point of view, the ECM morphology greatly influences the behaviour of cells by blocking and supporting their advancement. We compared our numerical model with experiments where endothelial cells migrate through the ECM from an endothelium formed in a side channel of the microfluidic system. In our model, we incorporated the ECM structure together with the chemotactic effect into the same parameter, describing the total angiogenic factor. With only one dynamic equation for our phase field, we can reproduce cellular migration and predict dynamics for the conformation of tip-like morphologies in the extracellular matrix. We also show that in our model, migrating cells adapt their shape to the morphology determined by the hydrogel while advancing towards the increasing chemotactic gradient. We have not included a proliferation term in our model; proliferation plays a significant role in collective cell dynamics at the lumen and capillary formation but not in endothelial tip advancement Siemerink et al. (2012); Yetkin-Arik et al. (2019).

We presented a fundamental dynamic model with a static environment and a dynamic model with extracellular matrix degradation for chemotactic cell migration. This novel model describes cell migration with a single equation and considers the extracellular matrix and chemotactic factors into a single parameter *C*_0_. This new consideration reduces parametrization while increasing the interpretability of the model. In the first case, the fundamental dynamic model, where cells are not allowed to degrade the ECM, endothelial cells migrate through the pore, avoiding the ECM and producing a tip and fingering morphology. Multiple finger-like morphologies and bifurcations will naturally emerge if the environment presents suitable conditions without introducing any additional rules. By introducing degradation and micro-environment sensing in our dynamic model with degradation, our cells migrated towards the general chemotactic gradient avoiding highly dense ECM regions and degrading the necessary ECM to self-generate a pore. In all cases, the model predicted realistic dynamics.

Our model of endothelial cell migration predicts tip velocities of the same order as in the experiments. Additionally, it can be used to predict the behaviour of directed migrating cells through different extracellular matrices and chemotactic factor distributions by simply modifying the value of the coupling parameter, *C*_0_. By including both the chemoattractants and the ECM in the model through this term, we can capture this complexity and provide a more accurate understanding of the dynamics of cell migration. This makes our model an excellent choice for testing and interpreting the behaviour of directed migrating cells in various microenvironments in laboratory experiments.

The model can be further improved, as this paper only shows the potential of the model to study the influence of the cellular environment on chemotactic cell migration. Our model opens a new framework to study cell migration interaction with different biomaterials. A simple way to extend the model would be to consider deformable extracellular matrices through a viscoelastic model to account for the rheology and deformations of the structure, leading to a more realistic dynamics representation.

## Data Availability

The raw data supporting the conclusion of this article will be made available by the authors, without undue reservation.
