# Supplementary material for: Modelling of chemotactic sprouting endothelial cells through an extracellular matrix
Source: Front Bioeng Biotechnol. 2023 Jun 8;11:1145550. doi: 10.3389/fbioe.2023.1145550 (PMC10285466; doi:10.3389/fbioe.2023.1145550)
Supplement: Supplementary file 1 [file DataSheet1.PDF]

frontiers

# *Supplementary Material*

## SUPPLEMENTARY DATA AND FIGURES

### Microfluidic assay and cell culture preparation

The experiments have been inspired by the microfluidic platform used in previously published experiments(López-Canosa et al., 2021, 2022). The microfluidic platform design (see Fig.1.A) from the main text) was performed using CAD software (AutoCAD 2019) and consists of a main cell culture chamber ($1300\mu m$ wide, $8800\mu m$ long, $150\mu m$ high) flanked by two media channels ($750\mu m$ wide and $150\mu m$ high). Two rows of 20 trapezoid microposts, each micropost with a large base of $300\mu m$, a small base of $70\mu m$ and a height of $200\mu m$, are located between the two mentioned structures. These trapezoids are $100\mu m$ apart and prevent the hydrogel from escaping into the lateral channels by confining it within the central chamber, thus facilitating continuous interaction between the cells and the hydrogel. See the trapezoid micropost design schema in Fig.S1. PDMS (Sylgard 184, Dow Corning) elastomer was mixed at a ratio of $10 : 1w/w$ (base: curing agent). After degassing and curing overnight at $65°C$, the PDMS was peeled off from the master, cut into individual devices, and punched. We made $6mm$ diameter holes for the media reservoirs and $1.2mm$ holes for the central chamber inlets. Finally, all the chips were thermally treated for 2h at $85°C$ to stabilize the bonding.

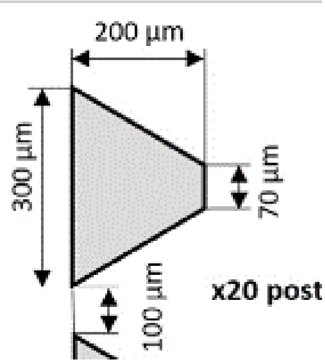


**Figure S1. Trapezoidal micropost schema.** The central chamber is delimited by two rows of 20 microposts, separated $100\mu m$.

All Bone marrow-derived rat endothelial progenitor cells (rEPCs) were obtained from long bones of young Lewis rats (2-4 weeks old) following methods following relevant guidelines and regulations, and all animal care protocols were approved by the Committee on Ethics, and Animal Experiments of the Scientific Park of Barcelona (Permit No. 0006S/13393/2011, 2011) (Aguirre et al., 2010; González-Vázquez et al., 2014). Immunofluorescence staining demonstrated an immature phenotype as endothelial progenitor cells and flow cytometry confirmed very low hematopoietic contamination in regular cultures. All these results were reported previously(Aguirre et al., 2010; López-Canosa et al., 2022). rEPC were resuspended at a density of $1 \cdot 10^6$ *cells/mL* and introduced into one of the side channels of the microfluidic platform. Then, we incubated the microfluidic platform for 45 minutes in an upright position to allow cell attachment to the central chamber side from the side channel. Following the incubation, we hydrate the other side channel by adding cell medium. Finally, the reservoirs are filled with medium at $120\mu L$ upstream and $90\mu L$ downstream and kept at $37°C$, $5\%CO_2$ incubator for the total duration of the experiment of 72h. The

desired VEGF concentration is introduced into the side-channel opposite to the endothelial cells at 24h after the incubation of the cells and maintained until the end of the experiment by refilling the reservoirs with the desired VEGF concentration. Endothelial cells are fixed after 72h with $4\%$ paraformaldehyde (Sigma Aldrich) for 15 min after washing cells with sterile PBS. The cells were permeabilized using a $0.5\%$ Triton X-110 solution before incubating the cells with phalloidin TRICT and DAPI.

The experimental verification of cell migration within the microfluidic system was evaluated through fluorescence microscopy. Cells were stained with F-actin (white) to capture the migration details, as shown in Fig.S2. The assay revealed the noticeable movement of the ECs in response to the VEGF concentration gradient, illustrating the successful application of our microfluidic platform for analysing cell migration behaviour. Image D (specifically labelled in the figure) was selected for comparison with our in-silico sprouting model results, bridging the gap between experimental observations and computational predictions.

Imaging is performed on a Leica TCS SP5 confocal microscope. The DAPI was excited with UV light at a laser power of $20\%$ and detected at a wavelength of 420-470 $nm$; TRICT phalloidin was excited with a wavelength of $561nm$ at a laser power of 20% and detected at a wavelength of 570-615$nm$.

## Fibrin hydrogel preparation

Fibrin hydrogel was prepared from commercial lyophilised fibrinogen (Sigma Aldrich, $> 75\%$) and bovine thrombin (Thermo Fisher Scientific, High purity grade). Fibrinogen ($2.5$ mg/mL) and thrombin ($100$ U/mL) solutions are prepared in Dulbecco's phosphate-buffered saline (DPBS) and mixed at a ratio of $100 : 2$ v/v (fibrinogen: thrombin).

## Characterisation of the hydrogel structure

Together with the cellular experiments, the fibrin hydrogel was characterised under the same conditions that prevail in the microfluidic chip. To study the structure of the fibrin hydrogel, fibrinogen is fluorescently labelled with NHS-rhodamine prior to polymerisation of the hydrogel. Hydrogel images, using confocal microscopy, are taken inside the microfluidic chip with the same polymerisation conditions to study conditions like cell culture. The fibrin hydrogel was exclusively introduced into the chip, devoid of any cellular or structural elements. Furthermore, the fibrinogen and thrombin were dissolved in PBS, precluding the labelling of any extraneous protein within the medium. This protocol ensured that all identified structures were exclusively associated with the hydrogel and did not encounter any interference. In these images, an irregular structure with high porosity can be observed. These images show a very fibrillar structure with a very open and interconnected skeleton, which favours the possibility of cells migrating through them. The pores are not very well defined and have irregular shapes and discontinuities in the walls, making it difficult to define a pore size. The porosity of the fibrin hydrogel network is assessed by analysing the acquired images, which involves calculating the void ratio through a threshold and estimating the pore dimensions via manual measurements of observed pores. It is determined that a fibrinogen hydrogel having a fibrinogen concentration of $2.5\ \frac{mg}{mL}$ and a thrombin content of $2\%$ by weight exhibits a void volume fraction of $75.37 \pm 1.29\%$ and a pore size of approximately $40\mu m$.

On the other hand, the fibrinogen hydrogel is analysed using a rheometer to study its mechanical properties. Viscosity, as well as $G'$ and $G''$, are analysed at $20°C$ (room temperature) and $37°C$ (cell growth temperature). It can be seen from the results that the fibrin hydrogel shows a shear thinning response, where the viscosity decreases with increasing shear stress. This non-Newtonian behaviour is also observed in other ECM derivatives such as collagen (Fernández-Pérez and Ahearne, 2019; Massensini et al., 2015).

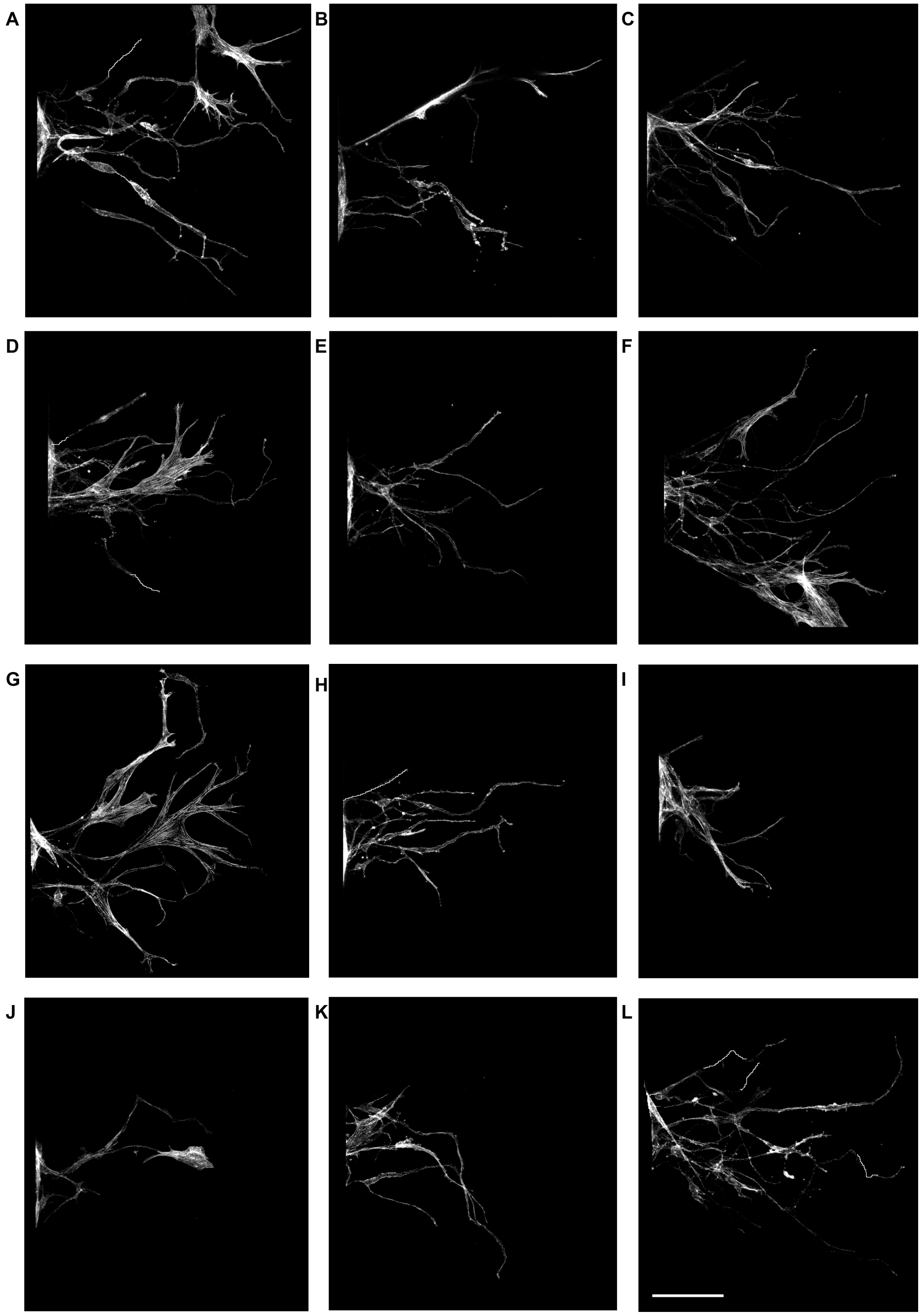


**Figure S2. Fluorescence microscopy images of the cell migration inside the microfluidic system after 48h under the influence of the angiogenic factor gradient.** The scale bar represents $100\mu m$. Cells were stained using F-actin (white).

If we compare the results for $20°C$ and $37°C$, we can see that the viscosity decreases with increasing temperature from $20°C$ to $37°C$, see Fig.S3 and Fig.S4. In addition, a storage modulus ($G'$) with a value around 200 Pa and a loss modulus ($G''$) with a value around $40Pa$ is obtained. These values of $G'$ are favourable for cell growth since it has been seen that cell viability is greater in hydrogels with a modulus of elasticity less than $1000Pa$(Asmani et al., 2013).

## Environment degradation and sensing

To introduce extracellular matrix (ECM) degradation, we use a Gaussian profile to generate the hole. For simplicity, we also consider that the MMPs degradation of the ECM is much faster than the tip advancement, so the presence of ECM does not hinder its progress.

At each time step, we compute the front position by localising the point in the middle of the most advanced interface layer, i.e. middle point of the $\min(y)$, where $\phi$ values $\in (-0.6, 0.6)$. With the tip located, we compute the sum of $C_0$ that lies in front of the tip at an approximately two-cell distance, i.e. $38.5\mu m$ or what is the same, two $5x5$ pixel matrices (above and below the tip). If the computed sum of the above matrix has a higher value, $\sum(C_0)^+ > \sum(C_0)^-$, then its centre moves 3 pixels upwards; it is lower, it moves 3 pixels downwards, and if it is equal, it stays on the same pixel. With this $\{+3, 0, -3\}$ pixel variation of the new centre, we place the degradation distribution (MMPs distribution) in front of the new centre. The MMPs distribution is considered a Gaussian-like distribution with a maximum value of 0.2 at its centre that expands up to 10 squared pixels. This maximum power of the Gaussian distribution is referred to as degradative power. Finally, we stop degradation, pixel-wise, when the hole generated by the MMPs reaches the same value as the chemotactic factor on the background.

# REFERENCES


Aguirre, A., González, A., Planell, J., and Engel, E. (2010). Extracellular calcium modulates in vitro bone marrow-derived flk-1+ cd34+ progenitor cell chemotaxis and differentiation through a calcium-sensing receptor. *Biochemical and biophysical research communications* 393, 156–161

Asmani, M. N., Ai, J., Amoabediny, G., Noroozi, A., Azami, M., Ebrahimi-Barough, S., et al. (2013). Three-dimensional culture of differentiated endometrial stromal cells to oligodendrocyte progenitor cells (opc s) in fibrin hydrogel. *Cell Biology International* 37, 1340–1349

Fernández-Pérez, J. and Ahearne, M. (2019). The impact of decellularization methods on extracellular matrix derived hydrogels. *Scientific Reports* 9, 1–12

González-Vázquez, A., Planell, J. A., and Engel, E. (2014). Extracellular calcium and casr drive osteoinduction in mesenchymal stromal cells. *Acta biomaterialia* 10, 2824–2833

López-Canosa, A., Pérez-Amodio, S., Engel, E., and Castaño, O. (2022). Microfluidic 3d platform to evaluate endothelial progenitor cell recruitment by bioactive materials. *Acta Biomaterialia*

López-Canosa, A., Perez-Amodio, S., Yanac-Huertas, E., Ordoño, J., Rodriguez-Trujillo, R., Samitier, J., et al. (2021). A microphysiological system combining electrospun fibers and electrical stimulation for the maturation of highly anisotropic cardiac tissue. *Biofabrication* 13, 035047

Massensini, A. R., Ghuman, H., Saldin, L. T., Medberry, C. J., Keane, T. J., Nicholls, F. J., et al. (2015). Concentration-dependent rheological properties of ecm hydrogel for intracerebral delivery to a stroke cavity. *Acta biomaterialia* 27, 116–130

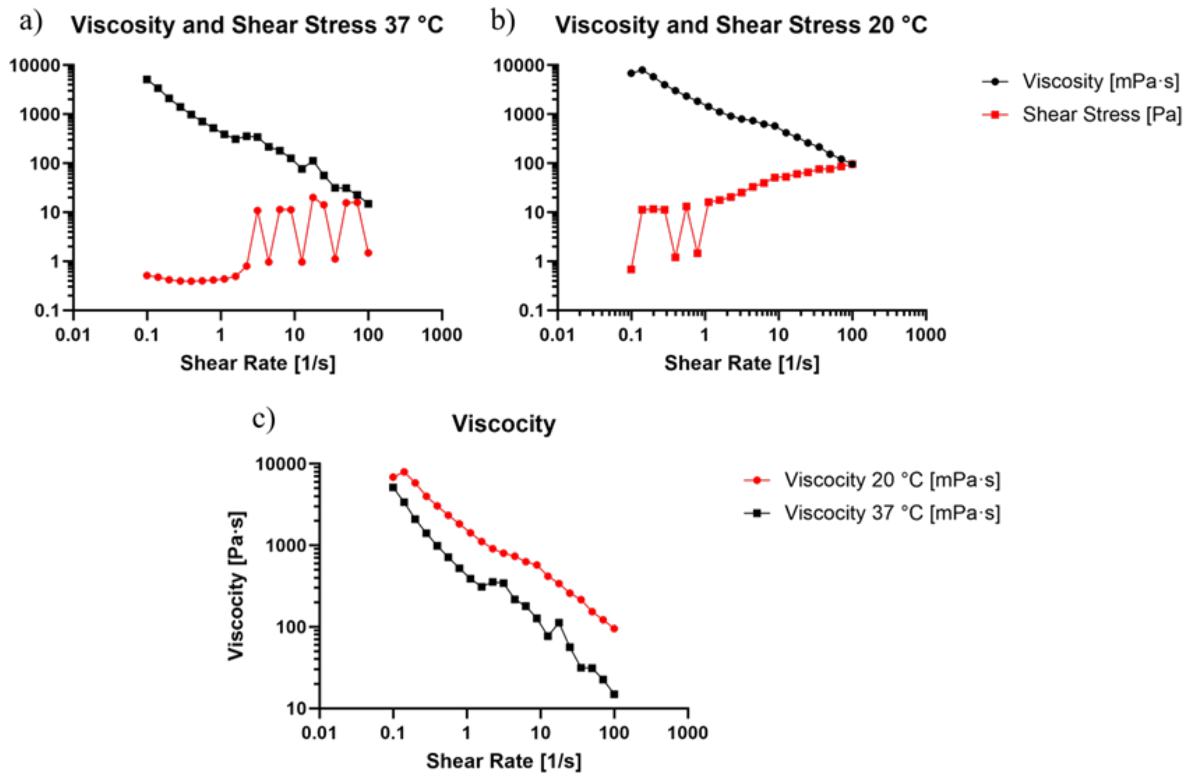


**Figure S3. Rheological analysis of fibrin hydrogel viscosity measurements at increasing shear rates.** **A)** Viscosity and shear stress at $37°C$ **B)** Viscosity and shear stress at 20 °C **C)** Viscosity comparison at different temperatures.

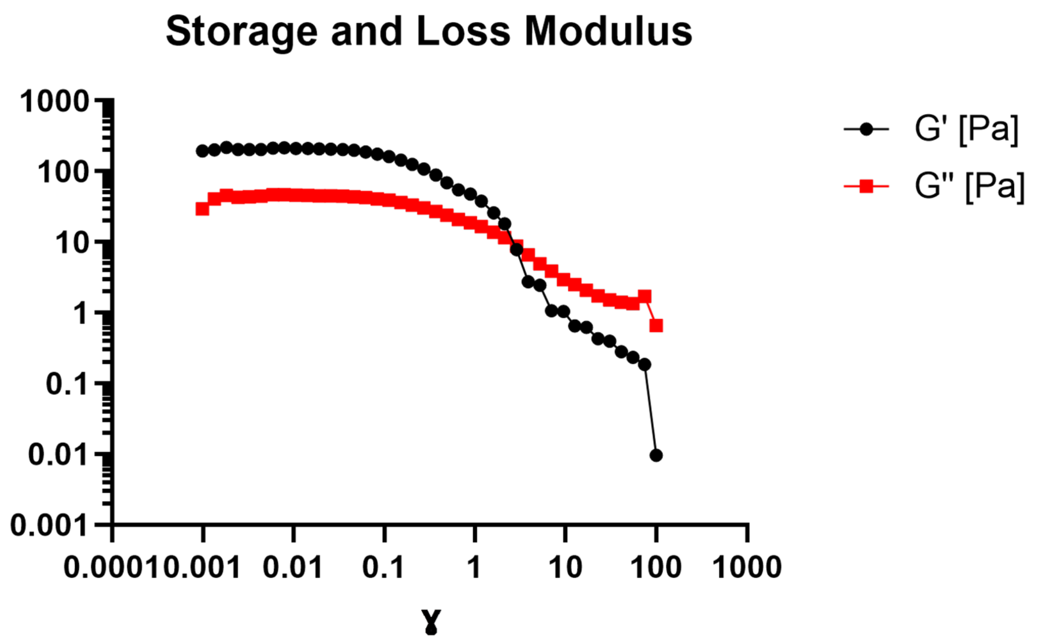


**Figure S4. Rheological analysis of measurements of storage modulus ($G'$) and loss modulus ($G''$) of fibrin hydrogel at increasing strain**.
